# A Study on the Optimal Machining Parameters of the Induction Assisted Milling with Inconel 718

**DOI:** 10.3390/ma12020233

**Published:** 2019-01-11

**Authors:** Eun Jung Kim, Choon Man Lee

**Affiliations:** Department of Mechanical Design and Manufacturing Engineering, School of Mechanical Engineering, Changwon National University, 20, Changwondaehak-ro, Uichang-gu, Changwon-si, Gyeongsangnam-do 51140, Korea; angel9940@hanmail.net

**Keywords:** induction assisted milling, tool wear, taguchi method, cutting tool

## Abstract

This paper focuses on an analysis of tool wear and optimum machining parameter in the induction assisted milling of Inconel 718 using high heat coated carbide and uncoated carbide tools. Thermally assisted machining is an effective machining method for difficult-to-cut materials such as nickel-based superalloy, titanium alloy, etc. Thermally assisted machining is a method of softening the workpiece by preheating using a heat source, such as a laser, plasma or induction heating. Induction assisted milling is a type of thermally assisted machining; induction preheating uses eddy-currents and magnetic force. Induction assisted milling has the advantages of being eco-friendly and economical. Additionally, the preheating temperature can be easily controlled. In this study, the Taguchi method is used to obtain the major parameters for the analysis of cutting force, surface roughness and tool wear of coated and uncoated tools under various machining conditions. Before machining experiments, a finite element analysis is performed to select the effective depth of the cut. The S/N ratio and ANOVA of the cutting force, surface roughness and tool wear are analyzed, and the response optimization method is used to suggest the optimal machining parameters.

## 1. Introduction

Recently, the production of difficult-to-cut materials has increased in various engineering industries. There are difficult-to-cut materials that have properties such as a high corrosion resistance, thermal resistance, good specific strength, etc. [1,2]. However, these materials are difficult to machine due to these very higher properties. Nickel-based super-alloys are metallic materials with a high temperature strength, toughness, and resistance to deterioration in corrosive or oxidizing environments [3,4,5].

Thermally assisted machining is a method of machining difficult-to-cut materials such as titanium alloys, nickel-based alloys and ceramics materials. According to the heat source type, machining can be classified into categories of induction assisted milling (IAM), laser-assisted milling (LAM), electrochemical assisted machining (ECAM) and plasma assisted milling (PAM). Thermally assisted machining uses heat sources to heat the workpiece and soften it. These machining methods have some advantages, such as a decrease in the cutting force and an improvement of surface roughness and energy saving compared to the conventional machining method [6,7,8,9,10].

However, tool wear is an issue that causes short tool life in the area of difficult-to-cut materials. Especially when machining nickel-based alloys, various types of tool wear are generated, such as mechanical wear, adhesive wear, abrasive wear, diffusion wear and oxidation wear. In conclusion, the tool life is shortened [11,12,13]. In order to reduce these problems, Pimenov examined the effect of the rate flank wear teeth face mills in the processing by using the regression analysis model [14]. Zhang et al. carried out a tool life and cutting force analysis with Inconel 718 under dry and minimum quantity cooling lubrication cutting condition [15]. Pimenov et al. carried out an automatic prediction study of required surface roughness by monitoring the wear in the face milling by using artificial intelligence methods [16].

However, research on optimal parameters has been lacking in the area of induction assisted milling. The selection of optimal machining parameters is an essential parameter for any process of difficult-to-cut machining. The selection of optimal parameters has an influence on the surface quality, the required cutting force and the cutting cost. Chamarthi et al. performed an investigation analysis of plasma arc cutting parameters such as voltage, cutting speed and plasma gas flow on a 12 mm hardox-400 plate workpiece [17]. Calleja et al. made improvements to the strategies and parameters for the multi-axis laser cladding operation [18]. Kim et al. predicted the cutting force and preheating-temperature for the laser-assisted milling of Inconel 718 and AISI 1045 steel and proposed effective machining conditions for the laser-assisted milling of Inconel 718 and AISI 1045 steels [19]. Venkatesan and Ramanujam took a statistical approach by using the multi-objective optimization method for the optimization of the influencing parameters in laser-assisted machining with the Inconel alloy [20]. Abbas et al. carried out a study on the minimization of turning time for high-strength steel with a given surface roughness using the Edgeworth–Pareto optimization method [21]. In this study, the response optimization method was used to determine the optimal machining parameters. This method is useful when evaluating the effect of multiple parameters on the response.

Due to its excellent abrasion and corrosion resistance, TiN coating is widely used to increase the cutting tool life. Al_2_O_3_ coating as a chemically inactive material has low thermal conductivity and excellent wear resistance. Additionally, it can function as a thermal diffusion barrier to increase the plastic deformation resistance. TiAlN or AlTiN (for aluminum contents higher than 50%) coating is aimed at the high-efficiency machining of difficult-to-cut materials, rather than at attempting to induce an increase in the tool life. In order to effectively perform dry machining, a coating tool is required to have a high oxidation resistance that can withstand the high-temperatures generated during machining. Additionally, a coating tool material is required to have excellent mechanical properties such as abrasion resistance and impact resistance [22]. Aslantas et al. carried out the analysis of the wear mechanism and tool life in turning hardened alloy steel by coated and uncoated Al_2_O_3_/TiCN mixed ceramic tools [23]. Liu et al. carried out a tool wear analysis in the high-speed machining of titanium alloys under dry and minimum quantity lubrication conditions by nc-AlTiN/a-Si_3_N_4_ and nc-AlCrN/a-Si_3_N_4_ coated tools. The results of the experiments were to propose dry and minimum quantity lubrication conditions according to the main wear typed for the two coated carbide tools [24].

In this study, an experiment was performed to determine the optimal machining parameters and to analyze the thermal effect and machinability of Inconel 718 under various milling conditions in the IAM. To investigate the thermal effects of tools and machinability in the IAM, the experiments were performed by using an AlTiN+HH (high heat special coating) coated carbide tool and of an uncoated carbide tool. The tool can be heated by an induction heat source, and the adhesive wear of the tool can be accelerated by the coating material. Therefore, the uncoated tool and the coated tool were compared to analyze the mechanism of tool wear by high-temperature induction assisted milling. Finite element analysis (FEA) was performed to find an effective depth of cut. The influence of the machining parameters on cutting force, surface roughness and tool wear was analyzed using the Taguchi method. The cutting force was measured by dynamometer and the surface roughness was measured by the shape measuring device. The tool wear was evaluated on the flank wear by microscope in accordance with ISO 8688-2: tool life testing in milling [25]. For the determination of the optimal machining parameters, the response optimization method was performed. An efficient machining condition to increase machinability was proposed and discussed.

## 2. Machining Method

### 2.1. Induction-Assisted Milling

**Figure 1:**
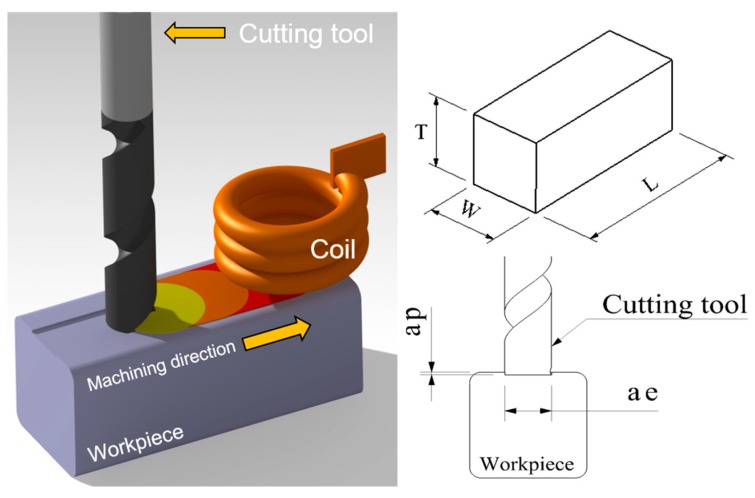
The schematic diagram of induction assisted milling.

Induction assisted milling is a type of thermally assisted machining that uses a heat source for induction. Thermally assisted machining is an effective machining method to improve machinability. It is a method by softening the workpiece by preheating using a heat source. The advantage of induction assisted heating is its clean, rapid heating, which makes it possible to accomplish uniform heating depending on the coil size; it also has an excellent thermal conduction loss and it is cheaper than other heating methods. The preheating temperature can be controlled by the number of coils, coil size and the frequency. Generally, an eddy-current loss has more influence than hysteresis in induction heating. When a material is heated, the contribution of hysteresis is usually very small so it can be ignored. In addition, induction heating is the ability to heat only a small portion of a workpiece and it is fast and clean, therefore, induction heating is a suitable heat source. In this study, one-way slot milling was used by using an 8 mm width of cut (tool diameter) under the various machining conditions. Figure 1 shows a schematic diagram of the induction assisted milling process.

### 2.2. Cutting Conditions

**Figure 2:**
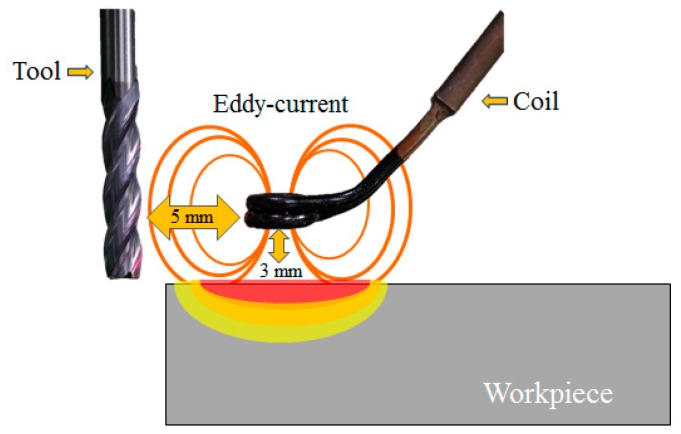
The distance between the tool, coil and workpiece.

**Figure 3:**
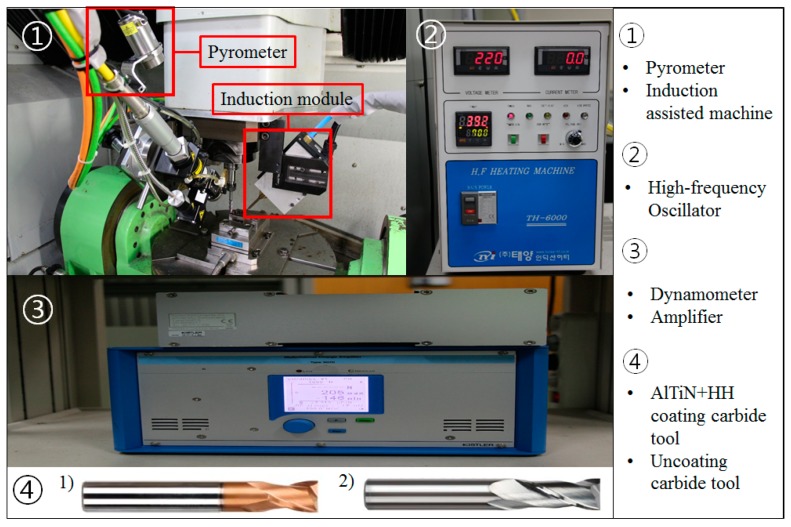
The experimental set-up.

The experiments use two types of cutting tools: (WIDIN Co., Ltd., Changwon, Korea, type ZE702080 and E302080) an AlTiN-HH (high heat special coating) coated carbide tool and an uncoated carbide tool. The cutting tool has the following specifications: diameter 8 mm, length 60 mm, and 2 flutes. The distance between the tool and the coil is 5 mm. Because the distance between the tool and the induction heat source is very close, the tool can be heated by an induction heat source. The distance between the coil and the workpiece is 3 mm. Because this distance between the coil and the workpiece is long, the preheating effect is low. Figure 2 shows the distance between the tool and the coil and the workpiece. This research was carried out on a 5-axis machining center (Hyundai-WIA Inc., Changwon, Korea, Hi-560M) and a 6 kW high-frequency induction heater module (Tae-yang Induction Heater Co., Ltd., Daegu, Korea, TH-6000). The pyrometer was installed under the laser preheating module fixed to a 5-axis machining center spindle. The temperature measurement range of the IR pyrometer (Process Sensors Co., Ltd., Milford, MA, USA, PSC-CS-Laser-2MH) was 385–1600 °C, the focal length was 1100 mm and the calibration of the pyrometer was carried out by identifying the target position with dual laser aiming. The measurement of the cutting force was done using a dynamometer and an amplifier (KISTLER Co., Ltd., Winterthur, Switzerland, 9257B, 5019). The surface roughness and tool wear were measured using a shape measurement device (OPTACOM GmbH & Co., Grettstadt, Germany, VC-10) and microscope (SOMETECH Inc., Seoul, Korea, IMS-345). Figure 3 and Table 1 show the experimental set-up and main cutting parameters.

## 3. Finite Element Analysis

### 3.1. Finite Element Analysis

Using the multi-physics ANSYS Electromagnetics and Workbench, finite element analyses were carried out to obtain the efficient depth of cut. The governing equation of the eddy current analysis can be expressed using Maxwell’s equations, as follows, Equation (1) [26].

(1) $$\nabla \times \left(\frac{1}{\mu_{0}\mu_{r}}\nabla \times A\right)+\sigma \frac{\partial A}{\partial t}-J_{s}=0$$

where A is the magnetic vector potential, $J_{s}$ is the source current density, $\mu_{0}$ is the vacuum magnetic permeability, $\mu_{r}$ is the relative magnetic permeability and σ is the electrical conductivity. The power of induction was 13 A, 380 V and the adaptive frequency was 300 kHz. These analysis conditions were determined by considering the specification of the induction oscillator. The method is an analyzing method by overlapping the heat source according to the feed rate [27]. The moving time of the heat source for the analysis was set to be 30 s by considering the lower feed rate (f = 100 mm/min) [28]. The material properties for the analysis are listed in Table 2. The chemical composition of Inconel 718 is as shown in Table 3. An electromagnetic analysis was performed first and then a temperature distribution was confirmed by thermal analysis.

### 3.2. Result of Analysis

**Figure 4:**
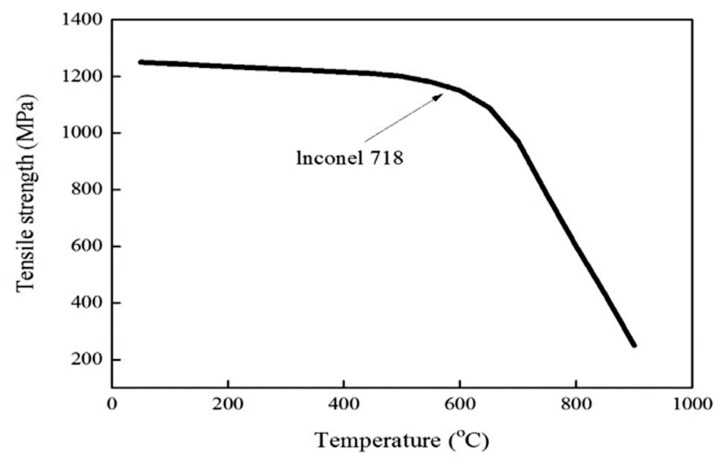
The tensile strength of Inconel 718 according to temperature (reproduced from [29], with permission from ELSEVIER, 2010).

**Figure 5:**
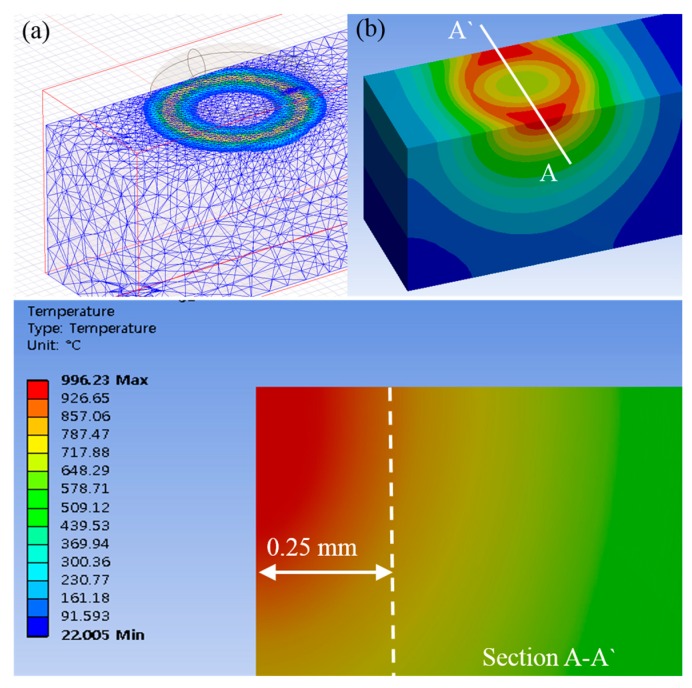
The results of finite element analysis; (**a**) electromagnetic, (**b**) thermal analysis.

When the preheating temperature is about 950 °C, the maximum depth of the cut is determined to be 0.25 mm by the finite element analysis, by considering the proper preheating effect, this could not be expected when the depth of the cut exceeds the maximum depth of the cut. The preheating temperature was chosen by considering the tensile strength of Inconel 718 according to temperature, as shown in Figure 4. Inconel 718 has its maximum elongation and minimum tensile strength at a temperature of about 950 °C. Additionally, Inconel 718 has a transformation point of its mechanical properties at about 700 °C. The temperature range of the effective depth of the cut is 700–950 °C. The depth of the cut range was determined within the maximum depth of cut of 0.25 mm by the finite element analysis. Figure 5 shows the finite element analysis results.

## 4. Experimental Set-Up

### 4.1. Taguchi Method

The Taguchi method, sometimes called the robust design method, is widely used to improve the quality of machining processes. The Taguchi design optimization method has three steps: system design, parameter design and tolerance design [30]. System design is the conceptual design level. Parameter design is the detailed design level, during which the nominal values of the design parameters and various dimensions need to be set. The advantage of the Taguchi method is that the exact choices of the values required are left undecided by the performance requirements of the system. This allows the parameters to be chosen so as to minimize the effects on the performance that arise from variations in the environment [31]. Many industrials or academic researchers have used the Taguchi method for optimization.

### 4.2. Experimental Design

Machining experiments were carried out using an Inconel 718 square workpiece. All experiments were repeated 3 times. The cutting parameters for the induction assisted milling operation are spindle speed (S), feed rate (F) and depth of cut (D). The spindle speed range (6000, 8000 and 10,000 rev/min) and the feed rate range (100, 150 and 200 mm/min) were selected after considering previous studies [9,32]. The depth of the cut range (0.15, 0.2 and 0.25 mm) with sufficient preheating effect was selected based on the finite element analysis result. The experimental design used an orthogonal array of the Taguchi method. The machining experiments were carried out according to the 3 levels and 3-factor L_9_ orthogonal array in Table 4.

## 5. Experimental Results and Discussion

**Figure 6:**
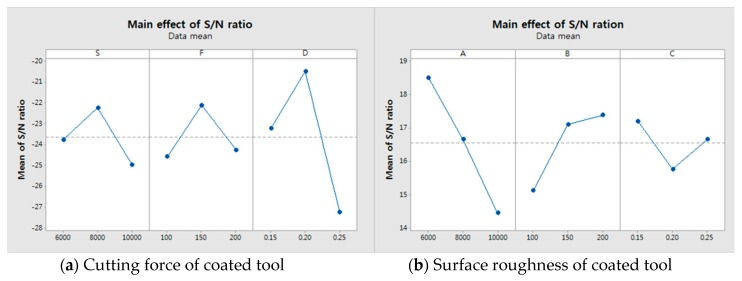

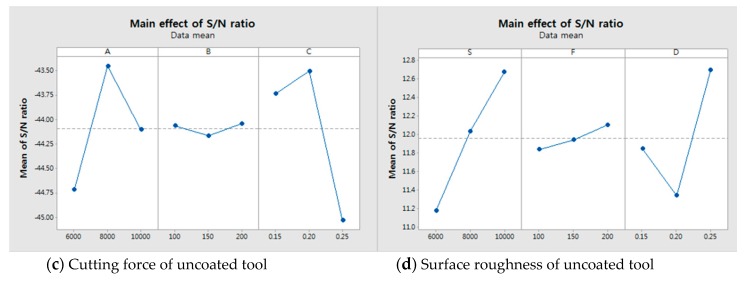
The main effect plot of the coated and uncoated tools on the cutting force and the surface roughness. (**a**) Cutting force of coated tool; (**b**) Surface roughness of coated tool; (**c**) Cutting force of uncoated tool; (**d**) Surface roughness of uncoated tool.

**Figure 7:**
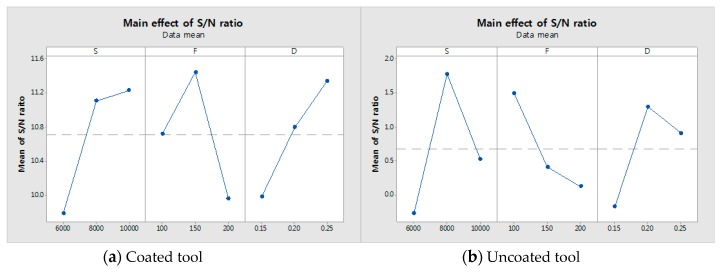
The main effect plot of the coated tool and uncoated tool on the tool wear. (**a**) Coated tool; (**b**) Uncoated tool.

The experimental results for cutting force, surface roughness, tool wear and the signal to noise (S/N) ratio are shown in Table 5, Table 6 and Table 7, for the coated tool and the uncoated tool. Figure 6 and Figure 7 show the main effect of S/N ratio of cutting force, surface roughness and tool wear. All the measurements were performed after machining for the same machining length. The cutting force was measured by the dynamometer and amplifier. The dynamometer was installed under the workpiece and fixture. The measured cutting force of the dynamometer is given by Equation (2),

(2) $$F=\sqrt{F_{x}{}^{2}+F_{y}{}^{2}}$$

where *Fx* is the tangential force and *Fy* is the radial force. A piezoelectric sensor was used for the dynamometer. The initial load was set to be zero. The surface roughness was measured by the shape measuring device. The surface roughness was measured as the arithmetic mean deviation of the profile (Ra) and 0.8 mm of the cut-off value, 4.5 mm of the evaluation length and a stylus tip radius of 2 mm. The evaluation length was analyzed repeatedly 5 times and the average value was selected as the surface roughness. The tool wear was measured by the flank wear using a microscope (SOMETECH Inc., Seoul, Korea, IMS-345). The evaluation of the tool wear was measured as well as the evaluated in the worst case in both flutes. The quality characteristics are classified into three types: nominal-is-best characteristics, larger-the-better characteristics and smaller-the-better characteristics. In this study, the smaller-the-better characteristics were used. The smaller-the-better characteristics are shown in Equation (3).

(3) $$S/N\;\mathrm{ratio}\;=\;-10\;\log\left(\Sigma \left(Y^{2}\right)/n\right)$$

where n is the number of observations and *Y* is the observed data.

### 5.1. S/N Ratio Analysis

According to the cutting force measurement results, the cutting force of the coated tool was approximately 1000% lower than that of the uncoated tool. The cutting force and the S/N ratio of the cutting force are shown in Table 5; the cutting force of the coated tool had the highest value of 28.62 N and the lowest value of 11.24 N. The uncoated tool had the highest value of 191.04 N and the lowest value of 138.86 N. Both the coated tool and the uncoated tool, for identical machining conditions, had highest values of S_1_F_3_D_3_ and lowest values of S_2_F_1_D_2_. For the coated and uncoated tools, the cutting force of the S/N ratio had the highest values of −21.73 dB and −43.45 dB. In the surface roughness measurement results, the surface roughness of the coated tool was found to be approximately 260% lower than that of the uncoated tool. The S/N ratio of the surface roughness is shown in Table 6; the surface roughness of the uncoated tool had its highest value at S_1_F_2_D_2_ (0.310 µm) and its lowest value at S_3_F_1_D_3_ (0.226 µm). The coated tool had its highest value at S_3_F_1_D_3_ (0.212 µm) and its lowest value at S_3_F_1_D_3_ (0.115 µm). The coated and uncoated tools had surface roughness S/N ratios that exhibited the highest values of 18.52 dB and 12.69 dB, respectively. For the tool wear measurement results, the tool wear of the coated tool was up to approximately 390% lower than that of the uncoated tool. The tool wear and S/N ratio of the tool wear are shown in Table 7. The tool wear of the coated tool had its highest value at S_2_F_3_D_1_ (0.340 mm) and its lowest value at S_1_F_3_D_3_ (1.075 mm). When the machining condition was S_2_F_1_D_2,_ the coated and uncoated tool to S/N ratio of tool wear had the highest values of 12.327 dB and 1.774 dB, respectively.

### 5.2. Main Effect Plot

We used the main effects plot to examine the differences between level means for one or more parameters. There is a main effect when different levels of a parameter affect the response differently. The larger the slope of the main effect plot is, the greater the magnitude of the main effect. Therefore, Figure 6 and Figure 7 show the main effects. The Figure 6a,b show main effects of S/N ratio in the cutting force and the surface roughness of coated tool. The Figure 6c,d show main effects of S/N ratio in the cutting force and the surface roughness of uncoated tool. The Figure 7a,b show main effects of S/N ratio in the tool wear of coated and uncoated tool. It has been determined from Figure 6a that the optimum levels were S_2_ (S:8000), F_2_ (f:150) and D_2_ (ap:0.2) and Figure 6b shows that the optimum levels were S_3_ (S:10,000), F_3_ (f:200) and D_3_ (ap:0.25). Figure 6c shows that the optimum levels were S_2_ (S:8000), F_3_ (f:200) and D_2_ (ap:0.2). Figure 6d shows that the optimum levels were S_1_ (S:6000), F_3_ (f:250) and D_1_ (ap:0.15). When comparing the coated tool with the uncoated tool, the cutting force shows a similar pattern but the surface roughness shows a different pattern. In the case of the tool wear, Figure 7a shows that the optimum level is S_3_F_2_D_3_, i.e., spindle speed is 10,000 rpm, the feed rate is 150 mm/min and the depth of cut is 0.25 mm. Figure 7b shows that the optimum level is S_2_F_1_D_2_, i.e., the spindle speed is 8000 rpm, the feed rate is 100 mm/min and the depth of cut is 0.2 mm.

### 5.3. Analysis of Variance

Analysis of Variance (ANOVA) is used to test the hypothesis that the means of two or more populations are equal. ANOVA assesses the importance of one or more parameters by comparing the response variable means at different parameter levels. ANOVA was performed to study the relative nature of the parameters. An ANOVA with 95% confidence was used. Table 8 shows the ANOVA results for cutting force, surface roughness and tool wear on the coated tool. It was observed that the depth of cut is the main contributing factor to the cutting force. The contributions of the cutting force were as follows: spindle speed at 19%, feed rate at 6% and depth of cut at 65%. It was observed that the spindle speed is the main contributing factor to the surface roughness. The contributions of the surface roughness were as follows: spindle speed at 59%, feed rate at 25% and depth of cut at 8%. It was observed that the spindle speed is the main contributing factor to tool wear. The contributions of tool wear were as follows: spindle speed at 37%, feed rate at 31% and depth of cut at 26%.

Table 9 shows the ANOVA results of cutting force, surface roughness and tool wear on the uncoated tool. It was observed that the depth of cut is the main contributing factor to the cutting force. The contributions to the cutting force were as follows: spindle speed at 35%, feed rate at 0.1% and depth of cut at 63%. It was observed that the spindle speed is the main contributing factor to the surface roughness. The contributions to the surface roughness were as follows: spindle speed at 48%, feed rate at 2% and depth of cut at 38%. It was observed that the spindle speed is the main contributing factor to the tool wear. The contributions to the tool wear were as follows: spindle speed at 48%, feed rate at 20% and depth of cut at 26%. In the contribution of the cutting force, the depth of cut has the most influence due to the decrease of the tool vibration according to the depth of heat affected zone. In the contributions of the surface roughness and tool wear, the coating blocked the heat transfer to the coated tool. So, the influence of the feed rate is increased in the coated tool. The F-test accurate only for normally distributed data. However, the three factors with three levels used in this study were not normally distributed. Therefore, the F-test was not suitable. We have performed the test for equation variance. It is possible for the null hypothesis of the variances to be equal. Table 10 shows the *p*-value of the test for equal variance.

### 5.4. Tool Wear and Machined Surface

**Figure 8:**
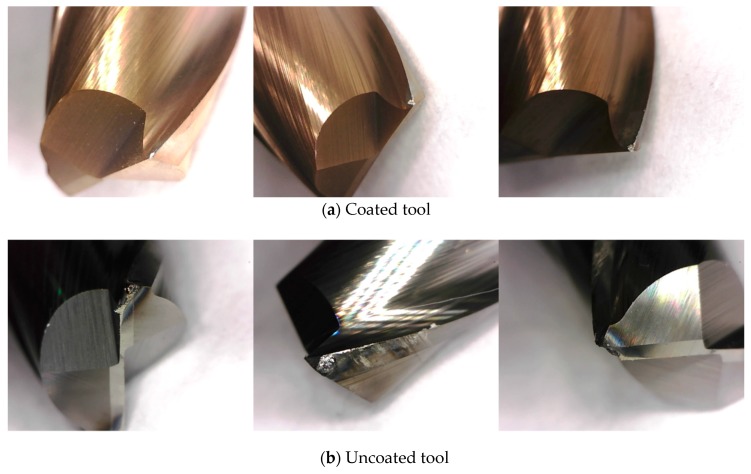
The tool wear of the coated tool and uncoated tool. (**a**) Coated tool; (**b**) Uncoated tool.

For the machining of Inconel 718 using carbide tools, diffusion loss occurs due to heat. Figure 8 shows the tool wear as found in the microscope measurement results. Figure 8 shows the measured parameters for level 7, i.e., the spindle speed is 10,000 rpm, the feed rate is 100 mm/min and the depth of cut is 0.25 mm both for the coated tool and the uncoated tool. The Figure 8a shows tool wear of coated tool and 8b shows tool wear of uncoated tool. Generally, the heat generated during machining is mainly transferred to the chip and cutting tool, and less than 10% of the generated heat is transferred to the workpiece. According to Figure 8a, the wear of the coated tool is insignificant. However, the uncoated tool is negatively influenced by the friction heat and radiant heat of the induction heat source. Figure 8b shows the worn tool after machining.

**Figure 9:**
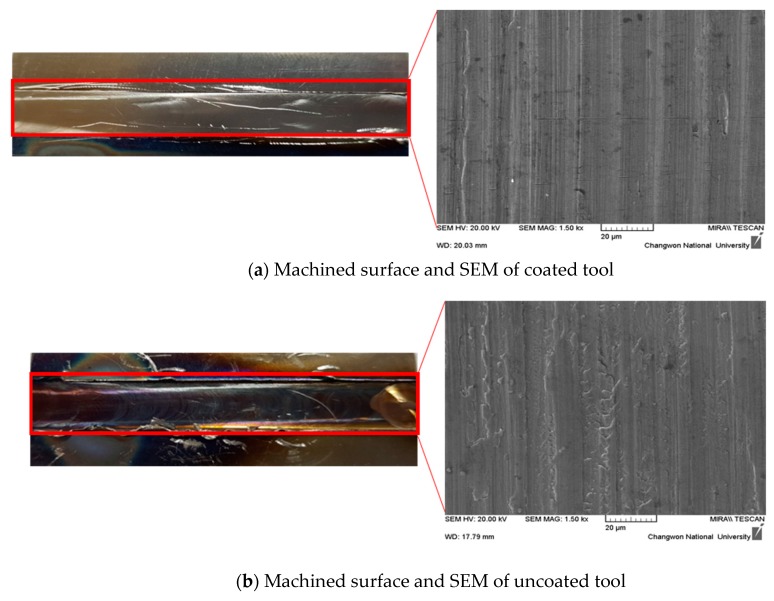
The machined surface and SEM of the coated tool and uncoated tool. (**a**) Machined surface and SEM of coated tool; (**b**) Machined surface and SEM of uncoated tool.

Figure 9a,b show the machined surfaces and scanning electron microscope of the coated and uncoated tools. When an AlTiN+HH coated tool is used in machining, the machined surface has no heat affected zone, as can be seen in Figure 9a. However, when the uncoated tool is used in machining, the machined surface has a heat affected zone, as can be seen in Figure 9b. The heat affected zone shows chemical and structural modifications. So, this phenomenon is very important in the area of thermally assisted machining. However, the uncoated tool has a rougher surface roughness than that of the coated tool, causing the uncoated tool to have a surface build up in a built-up-edge (BUE). When the uncoated tool was used, the heat generated during IAM was transferred to the workpiece more than to the coated tool. The analysis results of the machined surface showed that the coating was effectively blocked the heat generated during IAM.

### 5.5. Response Optimization

Based on the design of the experimental results, we carried out a response optimization. The goal was to achieve a minimum and weights are shown in Table 11. The result of the response optimization is depicted in Table 12. The parameters of the coated tool S_2_F_2_D_2_ were observed, i.e., a spindle speed if 8000 rpm, a feed rate of 150 mm/min, a depth of cut of 0.2 mm and a composite desirability of 0.78. Desirability assessed how well a combination of variables satisfies the goals defined for the response. The uncoated tool was S_2_F_1_D_2_, i.e., a spindle speed of 8000 rpm, a feed rate of 100 mm/min, a depth of cut of 0.2 mm and a composite desirability of 0.79. The coated tool and uncoated tool both have the same spindle speed and depth of cut. The coated tool had a higher feed rate than the uncoated tool. The machining time can be decreased by using the coated tool in IAM.

## 6. Conclusions

In this study, induction assisted milling (IAM) was carried out on Inconel 718 with an AlTiN coated carbide tool and uncoated carbide tool. The efficient depth of cut was selected using finite element analysis. The cutting force, surface roughness and tool wear were investigated using the Taguchi method for the determination of the optimal machining parameters. The findings of this study lead to the following conclusions:

1. Before the machining experiments, for an effective depth of cut determination, a multi-physics analysis by electromagnetic and thermal analyses was performed. When the preheating temperature is about 950 °C, the maximum depth of cut is determined to be 0.25 mm by the finite element analysis; the proper preheating effect could not be expected when the depth of cut exceeds the maximum depth of cut.
2. For the design of the experiments, a spindle speed of 8000 rpm, a feed rate of 150 mm/min and depth of cut 0.2 mm are the cutting parameters for the minimum cutting force for a coated tool and a spindle speed of 8000 rpm, a feed rate of 200 mm/min and depth of cut 0.2 mm are the cutting parameters for the minimum cutting force for an uncoated tool. The cutting force of the coated tool was lower than the uncoated tool. The surface roughness and tool wear, as the spindle speed increases, indicates the better quality on the coated tool.
3. Based on the ANOVA results, the main contributing factor for the cutting force of both the coated and uncoated tools were 65 and 63% depths of cut, respectively. The main contributing factor for the surface roughness of both of coated and uncoated tool was the spindle speed, 59 and 48%, respectively. The main contributing factor for tool wear of both the coated tool and uncoated tool was the spindle speed, which was approximately 37 and 48%, respectively. The uncoated tools have a lower machinability level due to the higher thermal effects by the heat source than coated tools.
4. The results of response optimization: the feed rate is different at the same conditions. A feed rate of 150 mm/min in the coated tool and a feed rate of 100 mm/min in the uncoated tool, a spindle speed of 8000 rpm and a depth of cut of 0.2 mm. An efficient preheating effect cannot be expected at a feed rate over the optimal feed rates in both tools. The coated tool has a higher feed rate due to the reduced thermal effect by the coating. The increase of feed rate can decrease the machining time and increase productivity. When the depth of cut of the analysis and the optimal parameter was compared, the FEA result showed a deeper response optimization due to the decrease of the preheating effect by the cooling and the moving.
5. Based on the machining experiment results, the uncoated tool was not recommended by induction assisted milling. The uncoated tool was affected by the thermal effect more than the coated tool, which can accelerate tool failure. The coated tool improved the tool wear up to a maximum of 390%. In the uncoated tool, the defects of the tools such as the fusion of chips, fracture and wear occurred due to thermal diffusion by the induction heat sources. During machining, the AlTiN+HH coating effectively reduces the heat generated by friction between the tool and the workpiece. The coated tool was demonstrated to be superior in efficiency for IAM compared to the uncoated tool.

## Figures and Tables

**Table 1:** The main cutting parameters.

Material	Inconel 718
Machining method	Slot milling
Material size (T × W × L, mm)	30 × 30 × 50
Depth of cut (ap, mm)	0.15, 0.2, 0.25
Width of cut (ae, mm)	8
Spindle speed (S, rev/min)	6000, 8000, 10,000
Feed rate (f, mm/min)	100, 150, 200
Feed per tooth (fz, mm/tooth)	0.008, 0.006, 0.005, 0.012, 0.009, 0.007, 0.016, 0.012, 0.010
Coated and uncoated tool	D8 Flat end-mill, 2F, 60L
Preheating temperature (°C)	950

**Table 2:** The material properties of Inconel 718.

Yield Strength (MPa)	Tensile Strength (MPa)	Elongation (%)	Hardness (HRC)
980	1280	12	42
Magnetic Permeability	Bulk conductivity (MS/m)	Specific heat (J/kg °C)	Thermal conductivity (W/mmK)
1.0011	80	435–637	11.2–25.8

**Table 3:** The chemical composition of Inconel 718.

Element	Ni	Cr	Nb	Mo	Ti	Al	Co	C	Mn	Si	Cu
wt%	50–55	17–21	4.75–5.5	2.8–3.3	0.65–1.15	0.2–0.8	≤1.0	≤0.08	≤0.35	≤0.35	≤0.3

**Table 4:** The experimental layout using an L_9_ orthogonal array.

Experimental No	S	f	ap
1	6000	100	0.15
2	6000	150	0.20
3	6000	200	0.25
4	8000	100	0.20
5	8000	150	0.25
6	8000	200	0.15
7	10,000	100	0.25
8	10,000	150	0.15
9	10,000	200	0.20

**Table 5:** The experimental results for cutting force and S/N ratio.

Experiment No	S	f	ap	Cutting Force (N)		S/N Ratio (dB)	
				Coated Tool	Uncoated Tool	Coated Tool	Uncoated Tool
1	1	1	1	15.16	164.88	−25.01	−44.72
2	1	2	2	13.23	161.90	−24.59	−44.06
3	1	3	3	28.10	191.04	−23.24	−43.73
4	2	1	2	11.24	138.86	−22.24	−43.45
5	2	2	3	15.14	167.41	−23.35	−44.16
6	2	3	1	12.72	141.66	−21.73	−43.50
7	3	1	3	28.62	177.76	−24.97	−44.10
8	3	2	1	15.88	155.40	−24.27	−44.04
9	3	3	2	12.23	149.20	−27.24	−45.03

**Table 6:** The experimental results for surface roughness and S/N ratio.

Experiment No	S	f	ap	Surface Roughness Ra (µm)		S/N Ratio (dB)	
				Coated Tool	Uncoated Tool	Coated Tool	Uncoated Tool
1	1	1	1	0.124	0.282	18.52	11.18
2	1	2	2	0.117	0.310	15.12	11.84
3	1	3	3	0.115	0.265	17.20	11.85
4	2	1	2	0.205	0.263	16.66	12.03
5	2	2	3	0.130	0.229	17.12	11.94
6	2	3	1	0.119	0.260	15.77	11.34
7	3	1	3	0.212	0.226	14.45	12.67
8	3	2	1	0.178	0.228	17.39	12.10
9	3	3	2	0.180	0.244	16.66	12.69

**Table 7:** The experimental results for tool wear and S/N ratio.

Experiment No	S	f	ap	Tool Wear (mm)		S/N Ratio (dB)	
				Coated Tool	Uncoated Tool	Coated Tool	Uncoated Tool
1	1	1	1	0.340	1.069	9.790	−0.272
2	1	2	2	0.304	0.956	10.718	1.492
3	1	3	3	0.329	1.075	11.206	−0.171
4	2	1	2	0.276	0.694	12.327	1.774
5	2	2	3	0.230	0.845	11.440	0.411
6	2	3	1	0.340	0.924	10.779	1.294
7	3	1	3	0.263	0.805	11.229	0.527
8	3	2	1	0.275	1.074	11.188	0.125
9	3	3	2	0.286	0.964	11.341	0.906

**Table 8:** The results of the ANOVA for cutting force, surface roughness, and tool wear on the coated tool.

Cutting Parameter	DOF	SS	MS	F	P	%C
Cutting force						
S	2	68.14	34.07	2.00	0.33	19.21
f	2	21.92	10.96	0.64	0.608	6.18
ap	2	230.63	115.32	6.77	0.129	65.01
Error	2	34.05	17.02			9.60
Total	8	354.74				100.00
Surface roughness						
S	2	0.007	0.003	10.14	0.09	59.75
f	2	0.003	0.001	4.37	0.186	25.78
ap	2	0.001	5.4E−0.4	1.45	0.407	8.58
Error	2	7.5E−0.4	3.7E−0.4			5.89
Total	8	0.012				100.00
Tool wear						
S	2	0.004	0.002	9.94	0.091	37.96
f	2	0.003	0.001	8.19	0.109	31.29
ap	2	0.003	0.001	7.05	0.124	26.94
Error	2	4.0E−4	2.1E−4			3.82
Total	8	0.011				100.00

**Table 9:** The results of the ANOVA for cutting force, surface roughness and tool wear on the uncoated tool.

Cutting Parameter	DOF	SS	MS	F	P	%C
Cutting force						
S	2	814.16	407.08	241.15	0.004	35.79
f	2	2.04	1.02	0.60	0.623	0.09
ap	2	1455.40	727.70	431.08	0.002	63.97
Error	2	3.38	1.688			0.15
Total	8	2274.98				100.00
Surface roughness						
S	2	0.003	0.001	4.39	0.077	48.17
f	2	1.0E−4	6.5E−5	0.19	0.99	2.04
ap	2	0.002	0.001	3.54	0.189	38.82
Error	2	7.0E−4				10.97
Total	8	0.006				100.00
Tool wear						
S	2	0.068	0.034	9.71	0.093	48.44
f	2	0.028	0.014	4.07	0.197	20.28
ap	2	0.037	0.018	5.27	0.159	26.29
Error	2	0.007	0.003			4.99
Total	8	0.141				100.00

**Table 10:** The test for the equal equation.

Parameter	Levene’s Test *p*-Value
Coated tool	0.009
Uncoated tool	0.001

**Table 11:** The response optimization.

Parameter	Goal	Target	Upper	Weight	Importance
Coated tool					
Cutting force	Minimum	11.240	28.62	1	1
Tool wear	Minimum	0.230	0.310	1	1
Surface roughness	Minimum	0.115	0.212	1	1
Uncoated tool					
Cutting force	Minimum	138.860	191.04	1	1
Tool wear	Minimum	0.694	1.075	1	1
Surface roughness	Minimum	0.226	0.310	1	1

**Table 12:** The response optimization results.

S	F	D	Cutting Force Optimization Plot	Tool Wear Optimization Plot	Surface Roughness Optimization Plot	Desirability
Coated tool						
8000	150	0.2	6.16	0.25	0.15	0.78
Uncoated tool						
8000	100	0.2	137.99	0.68	0.26	0.79

## Nomenclature

IAM	Induction assisted machining	*S*/*N*	Signal to noise
S	Spindle speed	OA	Orthogonal array
f	Feed rate	DOE	Design of experiments
ap	Depth of cut	DOF	Degree of freedom
ae	Width of cut	SS	Sum of squares
Fx	Tangential force	P	Probability value
Fy	Radial force	F	Fisher ration
T	Thickness	%C	Percentage contribution
W	Width	SS	Sum of squares
L	Length	MS	Mean of squares
fz	Feed per tooth	ANOVA	Analysis of variance
Ra	Arithmetic average roughness	dB	Decibel
SEM	Scanning electron microscope		
